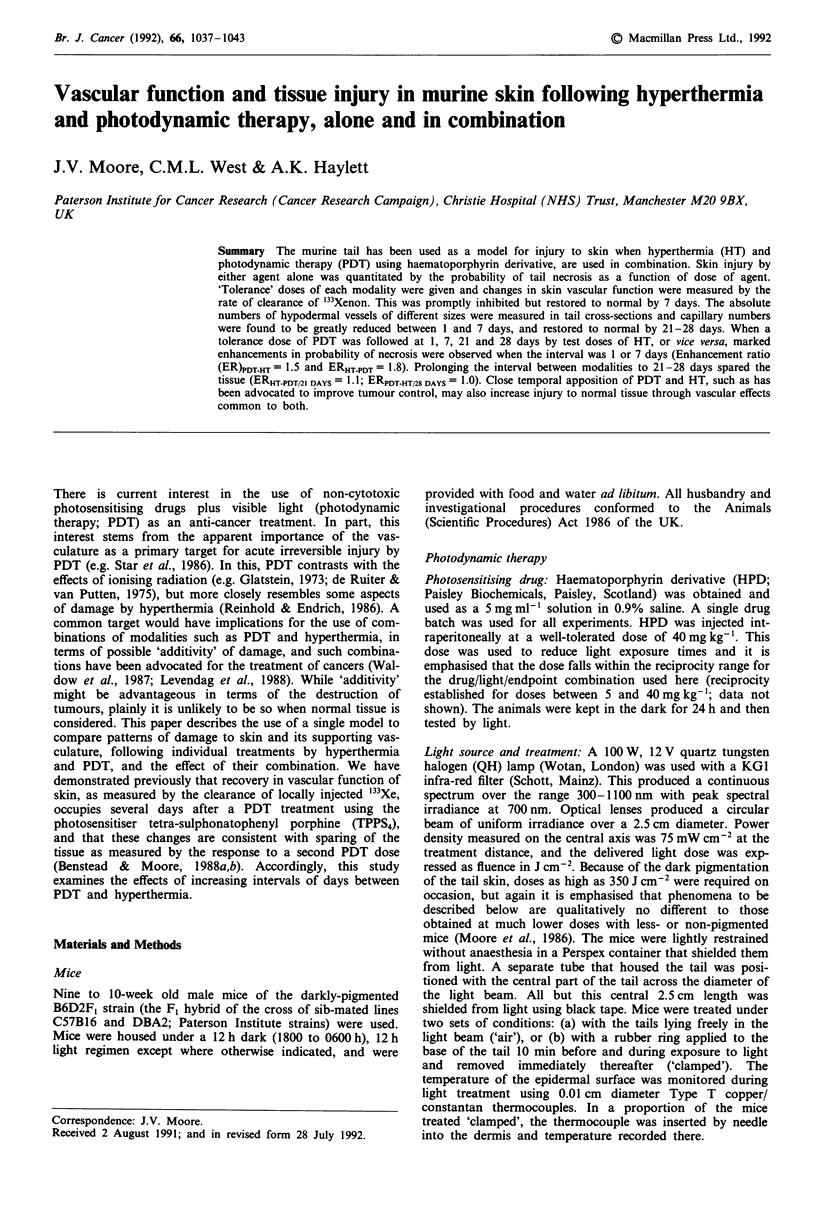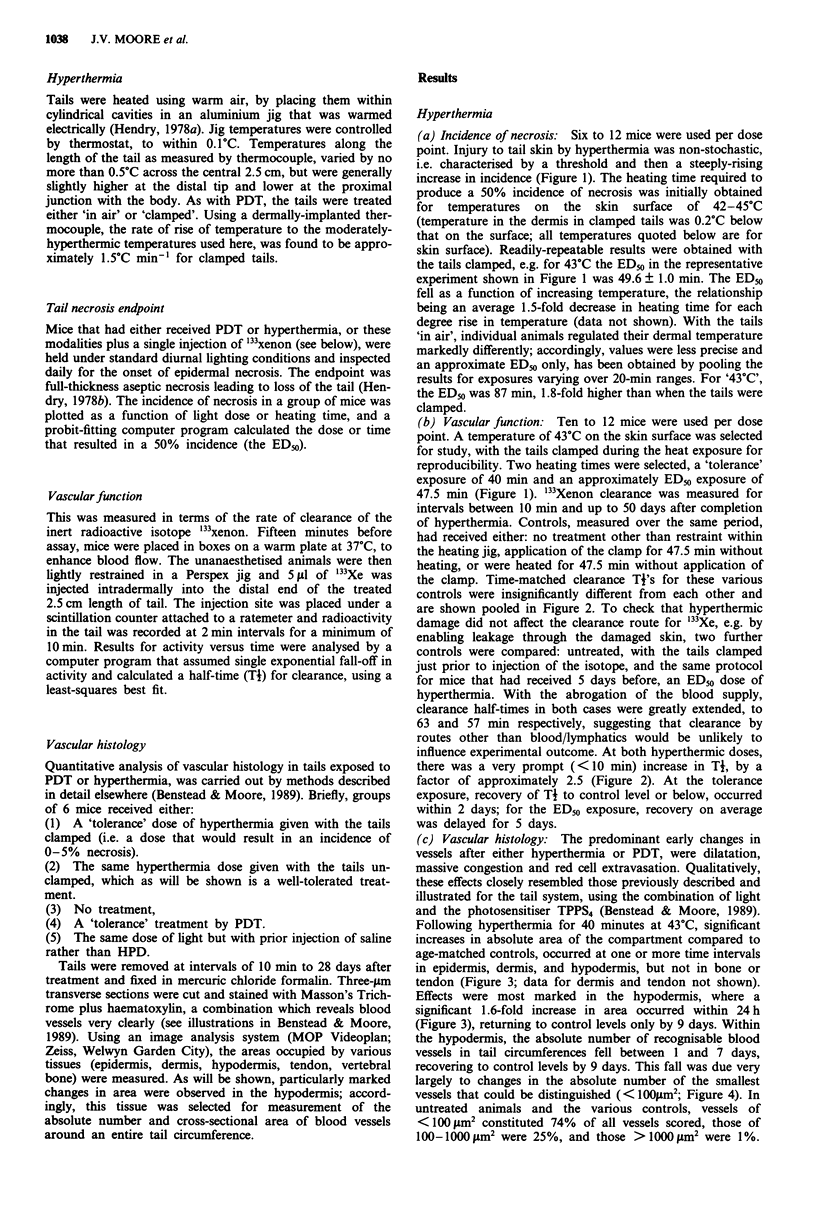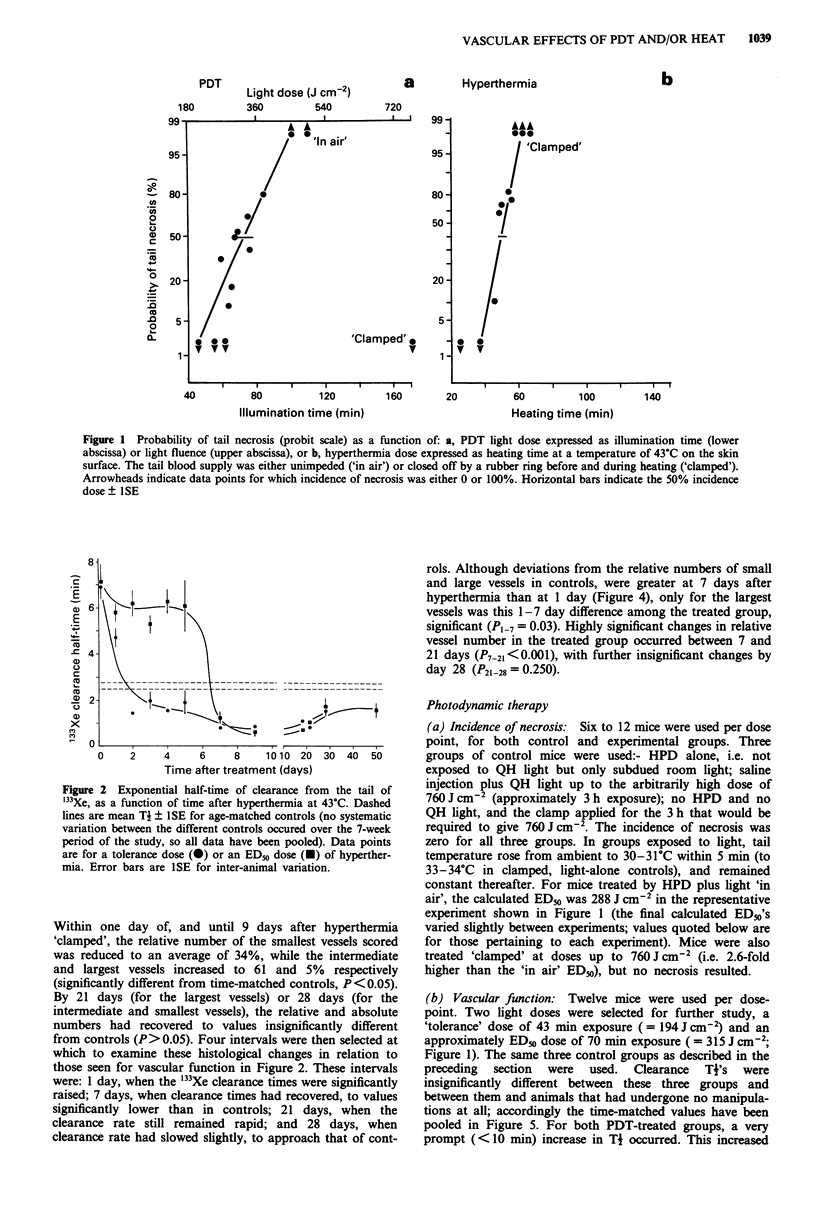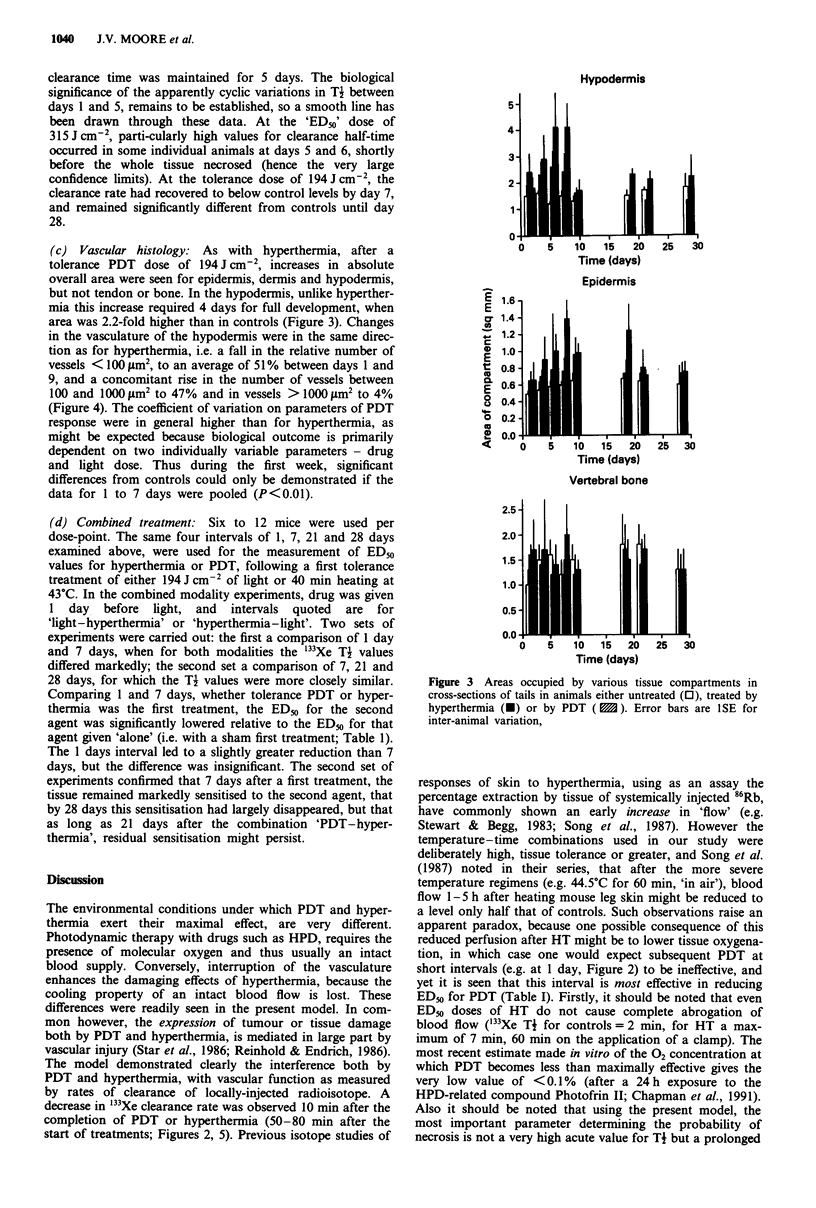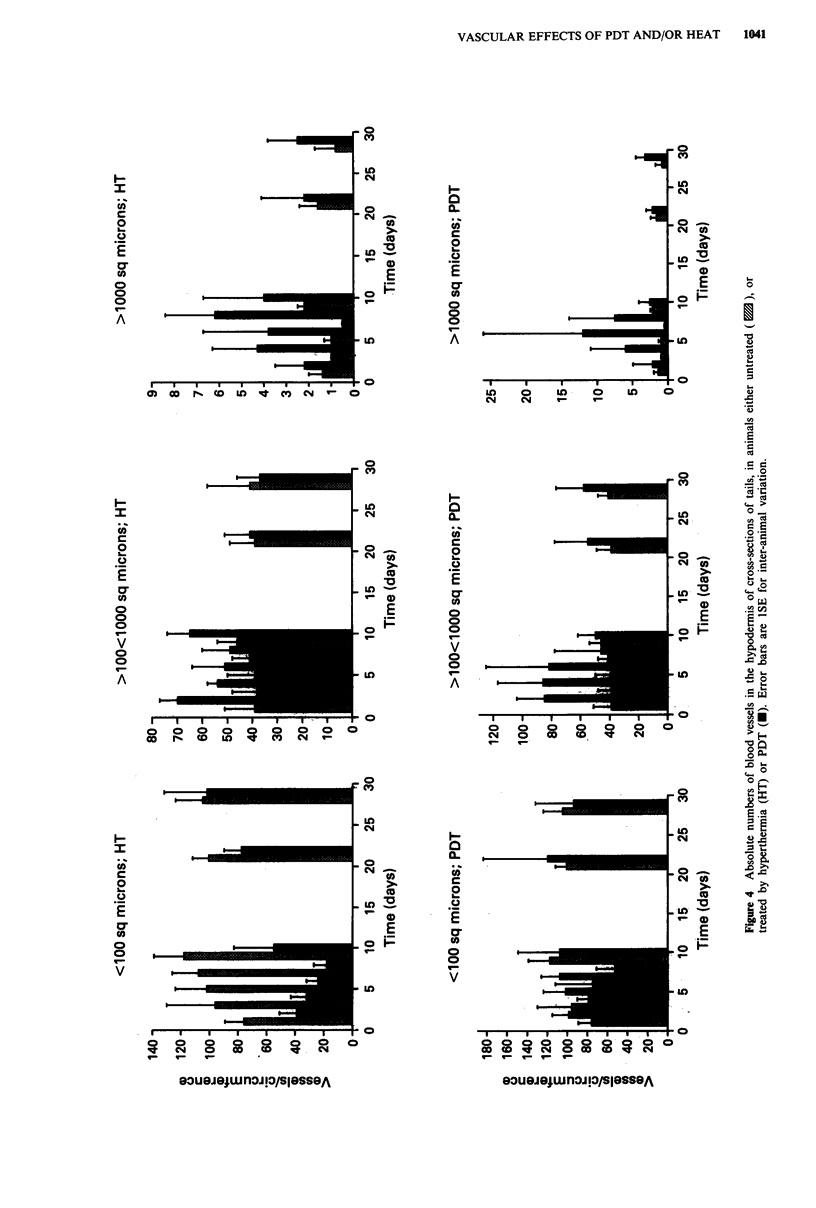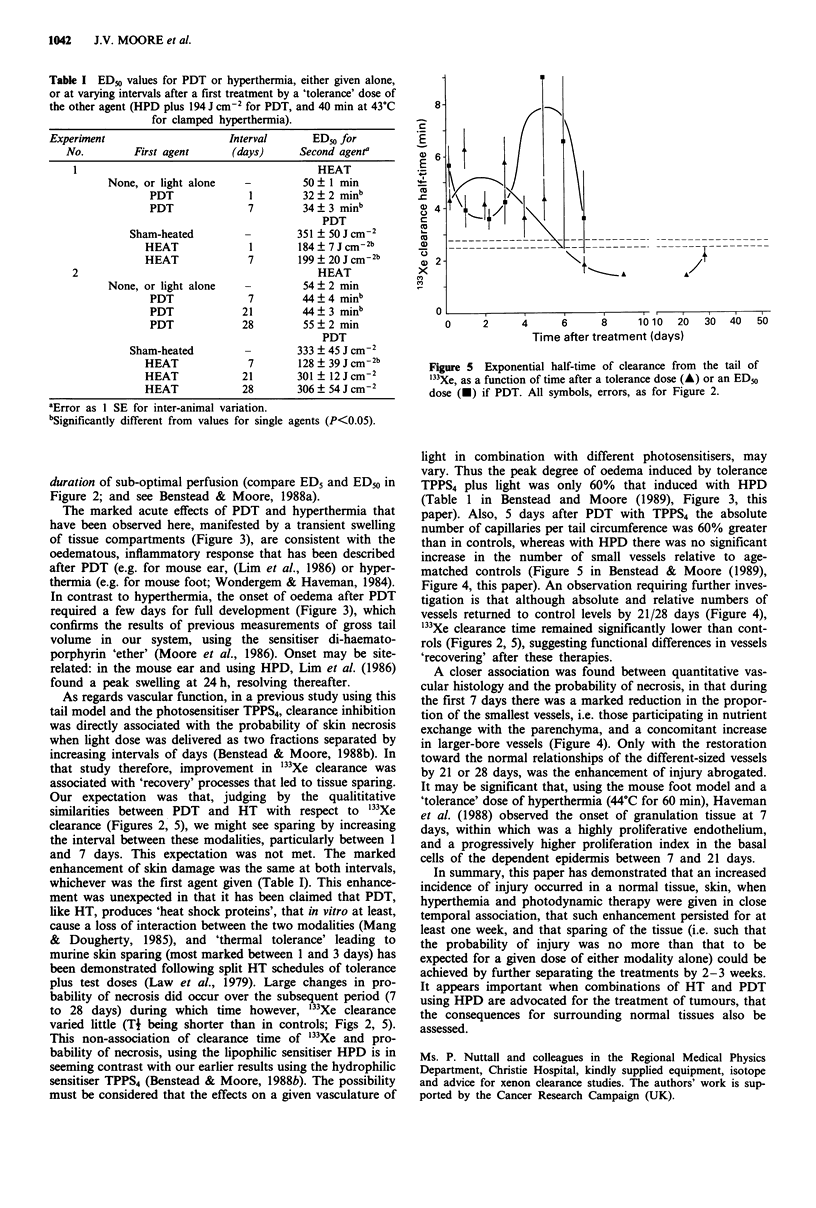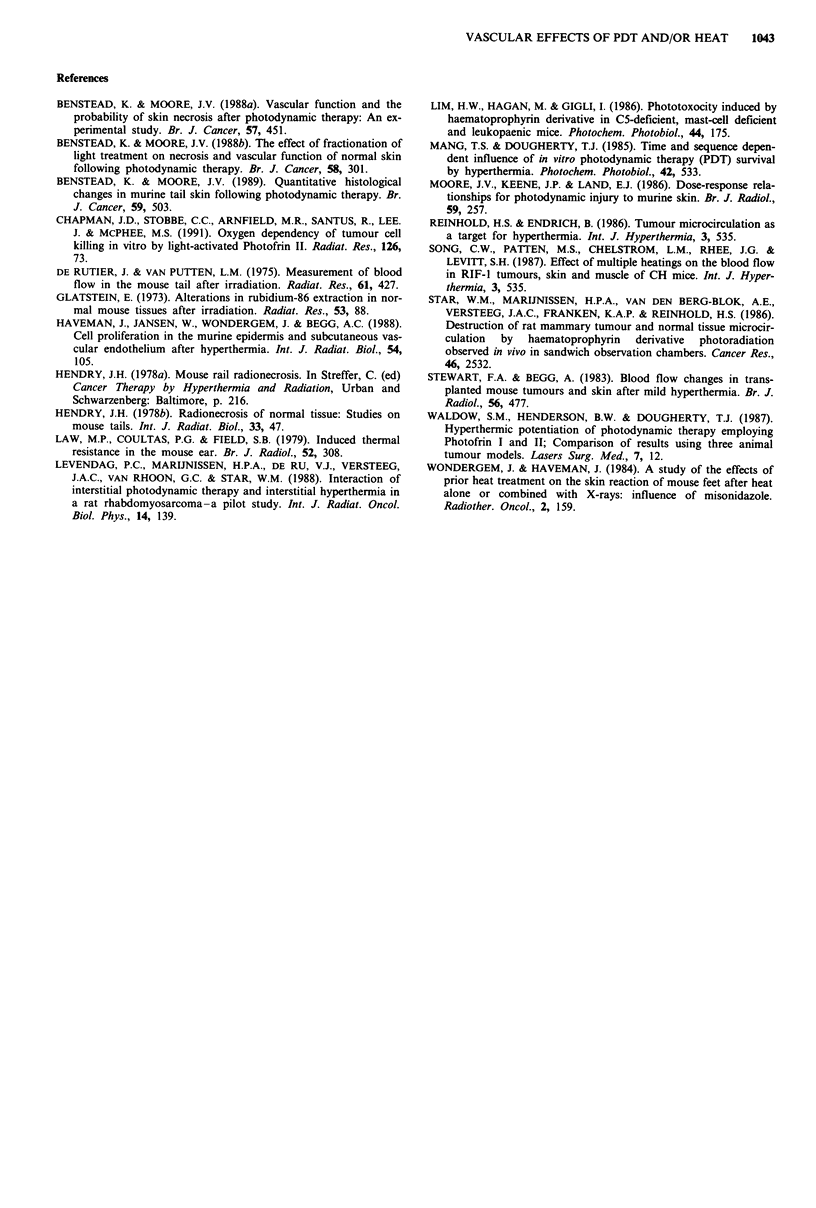# Vascular function and tissue injury in murine skin following hyperthermia and photodynamic therapy, alone and in combination.

**DOI:** 10.1038/bjc.1992.406

**Published:** 1992-12

**Authors:** J. V. Moore, C. M. West, A. K. Haylett

**Affiliations:** Paterson Institute for Cancer Research (Cancer Research Campaign), Christie Hospital (NHS) Trust, Manchester, UK.

## Abstract

The murine tail has been used as a model for injury to skin when hyperthermia (HT) and photodynamic therapy (PDT) using haematoporphyrin derivative, are used in combination. Skin injury by either agent alone was quantitated by the probability of tail necrosis as a function of dose of agent. 'Tolerance' doses of each modality were given and changes in skin vascular function were measured by the rate of clearance of 133Xenon. This was promptly inhibited but restored to normal by 7 days. The absolute numbers of hypodermal vessels of different sizes were measured in tail cross-sections and capillary numbers were found to be greatly reduced between 1 and 7 days, and restored to normal by 21-28 days. When a tolerance dose of PDT was followed at 1, 7, 21 and 28 days by test doses of HT, or vice versa, marked enhancements in probability of necrosis were observed when the interval was 1 or 7 days (Enhancement ratio (ER)PDT-HT = 1.5 and ERHT-PDT = 1.8). Prolonging the interval between modalities to 21-28 days spared the tissue (ERHT-PDT/21 DAYS = 1.1; ERPDT-HT/28 DAYS = 1.0). Close temporal apposition of PDT and HT, such as has been advocated to improve tumour control, may also increase injury to normal tissue through vascular effects common to both.


*Br. J. Cancer* (1992), **66**, 1037–1043 © Macmillan Press Ltd., 1992

### Vascular function and tissue injury in murine skin following hyperthermia and photodynamic therapy, alone and in combination

J.V. Moore, C.M.L. West & A.K. Haylett

*Paterson Institute for Cancer Research (Cancer Research Campaign), Christie Hospital (NHS) Trust, Manchester M20 9BX, UK*

**Summary** The murine tail has been used as a model for injury to skin when hyperthermia (HT) and photodynamic therapy (PDT) using haematoporphyrin derivative, are used in combination. Skin injury by either agent alone was quantitated by the probability of tail necrosis as a function of dose of agent. 'Tolerance' doses of each modality were given and changes in skin vascular function were measured by the rate of clearance of $^{133}$Xenon. This was promptly inhibited but restored to normal by 7 days. The absolute numbers of hypodermal vessels of different sizes were measured in tail cross-sections and capillary numbers were found to be greatly reduced between 1 and 7 days, and restored to normal by 21–28 days. When a tolerance dose of PDT was followed at 1, 7, 21 and 28 days by test doses of HT, or *vice versa*, marked enhancements in probability of necrosis were observed when the interval was 1 or 7 days (Enhancement ratio $(ER)_{PDT\text{-}HT} = 1.5$ and $ER_{HT\text{-}PDT} = 1.8$). Prolonging the interval between modalities to 21–28 days spared the tissue ($ER_{HT\text{-}PDT/21\ DAYS} = 1.1$; $ER_{PDT\text{-}HT/28\ DAYS} = 1.0$). Close temporal apposition of PDT and HT, such as has been advocated to improve tumour control, may also increase injury to normal tissue through vascular effects common to both.

There is current interest in the use of non-cytotoxic photosensitising drugs plus visible light (photodynamic therapy; PDT) as an anti-cancer treatment. In part, this interest stems from the apparent importance of the vasculature as a primary target for acute irreversible injury by PDT (e.g. Star *et al.*, 1986). In this, PDT contrasts with the effects of ionising radiation (e.g. Glatstein, 1973; de Ruiter & van Putten, 1975), but more closely resembles some aspects of damage by hyperthermia (Reinhold & Endrich, 1986). A common target would have implications for the use of combinations of modalities such as PDT and hyperthermia, in terms of possible 'additivity' of damage, and such combinations have been advocated for the treatment of cancers (Waldow *et al.*, 1987; Levendag *et al.*, 1988). While 'additivity' might be advantageous in terms of the destruction of tumours, plainly it is unlikely to be so when normal tissue is considered. This paper describes the use of a single model to compare patterns of damage to skin and its supporting vasculature, following individual treatments by hyperthermia and PDT, and the effect of their combination. We have demonstrated previously that recovery in vascular function of skin, as measured by the clearance of locally injected $^{133}$Xe, occupies several days after a PDT treatment using the photosensitiser tetra-sulphonatophenyl porphine ($TPPS_4$), and that these changes are consistent with sparing of the tissue as measured by the response to a second PDT dose (Benstead & Moore, 1988*a*,*b*). Accordingly, this study examines the effects of increasing intervals of days between PDT and hyperthermia.

### Materials and Methods

### *Mice*

Nine to 10-week old male mice of the darkly-pigmented $B6D2F_1$ strain (the $F_1$ hybrid of the cross of sib-mated lines C57B16 and DBA2; Paterson Institute strains) were used. Mice were housed under a 12 h dark (1800 to 0600 h), 12 h light regimen except where otherwise indicated, and were provided with food and water *ad libitum*. All husbandry and investigational procedures conformed to the Animals (Scientific Procedures) Act 1986 of the UK.

### *Photodynamic therapy*

*Photosensitising drug:* Haematoporphyrin derivative (HPD; Paisley Biochemicals, Paisley, Scotland) was obtained and used as a 5 mg ml$^{-1}$ solution in 0.9% saline. A single drug batch was used for all experiments. HPD was injected intraperitoneally at a well-tolerated dose of 40 mg kg$^{-1}$. This dose was used to reduce light exposure times and it is emphasised that the dose falls within the reciprocity range for the drug/light/endpoint combination used here (reciprocity established for doses between 5 and 40 mg kg$^{-1}$; data not shown). The animals were kept in the dark for 24 h and then tested by light.

*Light source and treatment:* A 100 W, 12 V quartz tungsten halogen (QH) lamp (Wotan, London) was used with a KG1 infra-red filter (Schott, Mainz). This produced a continuous spectrum over the range 300–1100 nm with peak spectral irradiance at 700 nm. Optical lenses produced a circular beam of uniform irradiance over a 2.5 cm diameter. Power density measured on the central axis was 75 mW cm$^{-2}$ at the treatment distance, and the delivered light dose was expressed as fluence in J cm$^{-2}$. Because of the dark pigmentation of the tail skin, doses as high as 350 J cm$^{-2}$ were required on occasion, but again it is emphasised that phenomena to be described below are qualitatively no different to those obtained at much lower doses with less- or non-pigmented mice (Moore *et al.*, 1986). The mice were lightly restrained without anaesthesia in a Perspex container that shielded them from light. A separate tube that housed the tail was positioned with the central part of the tail across the diameter of the light beam. All but this central 2.5 cm length was shielded from light using black tape. Mice were treated under two sets of conditions: (a) with the tails lying freely in the light beam ('air'), or (b) with a rubber ring applied to the base of the tail 10 min before and during exposure to light and removed immediately thereafter ('clamped'). The temperature of the epidermal surface was monitored during light treatment using 0.01 cm diameter Type T copper/constantan thermocouples. In a proportion of the mice treated 'clamped', the thermocouple was inserted by needle into the dermis and temperature recorded there.

Correspondence: J.V. Moore.
Received 2 August 1991; and in revised form 28 July 1992.

### Hyperthermia

Tails were heated using warm air, by placing them within cylindrical cavities in an aluminium jig that was warmed electrically (Hendry, 1978a). Jig temperatures were controlled by thermostat, to within 0.1°C. Temperatures along the length of the tail as measured by thermocouple, varied by no more than 0.5°C across the central 2.5 cm, but were generally slightly higher at the distal tip and lower at the proximal junction with the body. As with PDT, the tails were treated either 'in air' or 'clamped'. Using a dermally-implanted thermocouple, the rate of rise of temperature to the moderately-hyperthermic temperatures used here, was found to be approximately 1.5°C $min^{-1}$ for clamped tails.

### Tail necrosis endpoint

Mice that had either received PDT or hyperthermia, or these modalities plus a single injection of $^{133}$xenon (see below), were held under standard diurnal lighting conditions and inspected daily for the onset of epidermal necrosis. The endpoint was full-thickness aseptic necrosis leading to loss of the tail (Hendry, 1978b). The incidence of necrosis in a group of mice was plotted as a function of light dose or heating time, and a probit-fitting computer program calculated the dose or time that resulted in a 50% incidence (the $ED_{50}$).

### Vascular function

This was measured in terms of the rate of clearance of the inert radioactive isotope $^{133}$xenon. Fifteen minutes before assay, mice were placed in boxes on a warm plate at 37°C, to enhance blood flow. The unanaesthetised animals were then lightly restrained in a Perspex jig and 5 $\mu$l of $^{133}$Xe was injected intradermally into the distal end of the treated 2.5 cm length of tail. The injection site was placed under a scintillation counter attached to a ratemeter and radioactivity in the tail was recorded at 2 min intervals for a minimum of 10 min. Results for activity versus time were analysed by a computer program that assumed single exponential fall-off in activity and calculated a half-time ($T\frac{1}{2}$) for clearance, using a least-squares best fit.

### Vascular histology

Quantitative analysis of vascular histology in tails exposed to PDT or hyperthermia, was carried out by methods described in detail elsewhere (Benstead & Moore, 1989). Briefly, groups of 6 mice received either:

(1) A 'tolerance' dose of hyperthermia given with the tails clamped (i.e. a dose that would result in an incidence of 0–5% necrosis).
(2) The same hyperthermia dose given with the tails unclamped, which as will be shown is a well-tolerated treatment.
(3) No treatment,
(4) A 'tolerance' treatment by PDT.
(5) The same dose of light but with prior injection of saline rather than HPD.

Tails were removed at intervals of 10 min to 28 days after treatment and fixed in mercuric chloride formalin. Three-$\mu$m transverse sections were cut and stained with Masson's Trichrome plus haematoxylin, a combination which reveals blood vessels very clearly (see illustrations in Benstead & Moore, 1989). Using an image analysis system (MOP Videoplan; Zeiss, Welwyn Garden City), the areas occupied by various tissues (epidermis, dermis, hypodermis, tendon, vertebral bone) were measured. As will be shown, particularly marked changes in area were observed in the hypodermis; accordingly, this tissue was selected for measurement of the absolute number and cross-sectional area of blood vessels around an entire tail circumference.

### Results

### Hyperthermia

*(a) Incidence of necrosis:* Six to 12 mice were used per dose point. Injury to tail skin by hyperthermia was non-stochastic, i.e. characterised by a threshold and then a steeply-rising increase in incidence (Figure 1). The heating time required to produce a 50% incidence of necrosis was initially obtained for temperatures on the skin surface of 42–45°C (temperature in the dermis in clamped tails was 0.2°C below that on the surface; all temperatures quoted below are for skin surface). Readily-repeatable results were obtained with the tails clamped, e.g. for 43°C the $ED_{50}$ in the representative experiment shown in Figure 1 was 49.6 ± 1.0 min. The $ED_{50}$ fell as a function of increasing temperature, the relationship being an average 1.5-fold decrease in heating time for each degree rise in temperature (data not shown). With the tails 'in air', individual animals regulated their dermal temperature markedly differently; accordingly, values were less precise and an approximate $ED_{50}$ only, has been obtained by pooling the results for exposures varying over 20-min ranges. For '43°C', the $ED_{50}$ was 87 min, 1.8-fold higher than when the tails were clamped.

*(b) Vascular function:* Ten to 12 mice were used per dose point. A temperature of 43°C on the skin surface was selected for study, with the tails clamped during the heat exposure for reproducibility. Two heating times were selected, a 'tolerance' exposure of 40 min and an approximately $ED_{50}$ exposure of 47.5 min (Figure 1). $^{133}$Xenon clearance was measured for intervals between 10 min and up to 50 days after completion of hyperthermia. Controls, measured over the same period, had received either: no treatment other than restraint within the heating jig, application of the clamp for 47.5 min without heating, or were heated for 47.5 min without application of the clamp. Time-matched clearance $T\frac{1}{2}$'s for these various controls were insignificantly different from each other and are shown pooled in Figure 2. To check that hyperthermic damage did not affect the clearance route for $^{133}$Xe, e.g. by enabling leakage through the damaged skin, two further controls were compared: untreated, with the tails clamped just prior to injection of the isotope, and the same protocol for mice that had received 5 days before, an $ED_{50}$ dose of hyperthermia. With the abrogation of the blood supply, clearance half-times in both cases were greatly extended, to 63 and 57 min respectively, suggesting that clearance by routes other than blood/lymphatics would be unlikely to influence experimental outcome. At both hyperthermic doses, there was a very prompt ($<$10 min) increase in $T\frac{1}{2}$, by a factor of approximately 2.5 (Figure 2). At the tolerance exposure, recovery of $T\frac{1}{2}$ to control level or below, occurred within 2 days; for the $ED_{50}$ exposure, recovery on average was delayed for 5 days.

*(c) Vascular histology:* The predominant early changes in vessels after either hyperthermia or PDT, were dilatation, massive congestion and red cell extravasation. Qualitatively, these effects closely resembled those previously described and illustrated for the tail system, using the combination of light and the photosensitiser $TPPS_4$ (Benstead & Moore, 1989). Following hyperthermia for 40 minutes at 43°C, significant increases in absolute area of the compartment compared to age-matched controls, occurred at one or more time intervals in epidermis, dermis, and hypodermis, but not in bone or tendon (Figure 3; data for dermis and tendon not shown). Effects were most marked in the hypodermis, where a significant 1.6-fold increase in area occurred within 24 h (Figure 3), returning to control levels only by 9 days. Within the hypodermis, the absolute number of recognisable blood vessels in tail circumferences fell between 1 and 7 days, recovering to control levels by 9 days. This fall was due very largely to changes in the absolute number of the smallest vessels that could be distinguished ($<100 \mu m^2$; Figure 4). In untreated animals and the various controls, vessels of $<100 \mu m^2$ constituted 74% of all vessels scored, those of $100-1000 \mu m^2$ were 25%, and those $>1000 \mu m^2$ were 1%.

**Figure 1** Probability of tail necrosis (probit scale) as a function of: **a**, PDT light dose expressed as illumination time (lower abscissa) or light fluence (upper abscissa), or **b**, hyperthermia dose expressed as heating time at a temperature of 43°C on the skin surface. The tail blood supply was either unimpeded ('in air') or closed off by a rubber ring before and during heating ('clamped'). Arrowheads indicate data points for which incidence of necrosis was either 0 or 100%. Horizontal bars indicate the 50% incidence dose ± 1SE

**Figure 2** Exponential half-time of clearance from the tail of $^{133}$Xe, as a function of time after hyperthermia at 43°C. Dashed lines are mean T½ ± 1SE for age-matched controls (no systematic variation between the different controls occured over the 7-week period of the study, so all data have been pooled). Data points are for a tolerance dose (●) or an $ED_{50}$ dose (■) of hyperthermia. Error bars are 1SE for inter-animal variation.

Within one day of, and until 9 days after hyperthermia 'clamped', the relative number of the smallest vessels scored was reduced to an average of 34%, while the intermediate and largest vessels increased to 61 and 5% respectively (significantly different from time-matched controls, $P<0.05$). By 21 days (for the largest vessels) or 28 days (for the intermediate and smallest vessels), the relative and absolute numbers had recovered to values insignificantly different from controls ($P>0.05$). Four intervals were then selected at which to examine these histological changes in relation to those seen for vascular function in Figure 2. These intervals were: 1 day, when the $^{133}$Xe clearance times were significantly raised; 7 days, when clearance times had recovered, to values significantly lower than in controls; 21 days, when the clearance rate still remained rapid; and 28 days, when clearance rate had slowed slightly, to approach that of controls. Although deviations from the relative numbers of small and large vessels in controls, were greater at 7 days after hyperthermia than at 1 day (Figure 4), only for the largest vessels was this 1–7 day difference among the treated group, significant ($P_{1-7}=0.03$). Highly significant changes in relative vessel number in the treated group occurred between 7 and 21 days ($P_{7-21}<0.001$), with further insignificant changes by day 28 ($P_{21-28}=0.250$).

### *Photodynamic therapy*

*(a) Incidence of necrosis:* Six to 12 mice were used per dose point, for both control and experimental groups. Three groups of control mice were used:- HPD alone, i.e. not exposed to QH light but only subdued room light; saline injection plus QH light up to the arbitrarily high dose of 760 J cm$^{-2}$ (approximately 3 h exposure); no HPD and no QH light, and the clamp applied for the 3 h that would be required to give 760 J cm$^{-2}$. The incidence of necrosis was zero for all three groups. In groups exposed to light, tail temperature rose from ambient to 30–31°C within 5 min (to 33–34°C in clamped, light-alone controls), and remained constant thereafter. For mice treated by HPD plus light 'in air', the calculated $ED_{50}$ was 288 J cm$^{-2}$ in the representative experiment shown in Figure 1 (the final calculated $ED_{50}$'s varied slightly between experiments; values quoted below are for those pertaining to each experiment). Mice were also treated 'clamped' at doses up to 760 J cm$^{-2}$ (i.e. 2.6-fold higher than the 'in air' $ED_{50}$), but no necrosis resulted.

*(b) Vascular function:* Twelve mice were used per dose-point. Two light doses were selected for further study, a 'tolerance' dose of 43 min exposure ( = 194 J cm$^{-2}$) and an approximately $ED_{50}$ dose of 70 min exposure ( = 315 J cm$^{-2}$; Figure 1). The same three control groups as described in the preceding section were used. Clearance T½'s were insignificantly different between these three groups and between them and animals that had undergone no manipulations at all; accordingly the time-matched values have been pooled in Figure 5. For both PDT-treated groups, a very prompt (<10 min) increase in T½ occurred. This increased clearance time was maintained for 5 days. The biological significance of the apparently cyclic variations in T½ between days 1 and 5, remains to be established, so a smooth line has been drawn through these data. At the '$ED_{50}$' dose of 315 J $cm^{-2}$, parti-cularly high values for clearance half-time occurred in some individual animals at days 5 and 6, shortly before the whole tissue necrosed (hence the very large confidence limits). At the tolerance dose of 194 J $cm^{-2}$, the clearance rate had recovered to below control levels by day 7, and remained significantly different from controls until day 28.

*(c) Vascular histology:* As with hyperthermia, after a tolerance PDT dose of 194 J $cm^{-2}$, increases in absolute overall area were seen for epidermis, dermis and hypodermis, but not tendon or bone. In the hypodermis, unlike hyperthermia this increase required 4 days for full development, when area was 2.2-fold higher than in controls (Figure 3). Changes in the vasculature of the hypodermis were in the same direction as for hyperthermia, i.e. a fall in the relative number of vessels $< 100\ \mu m^2$, to an average of 51% between days 1 and 9, and a concomitant rise in the number of vessels between 100 and 1000 $\mu m^2$ to 47% and in vessels $> 1000\ \mu m^2$ to 4% (Figure 4). The coefficient of variation on parameters of PDT response were in general higher than for hyperthermia, as might be expected because biological outcome is primarily dependent on two individually variable parameters – drug and light dose. Thus during the first week, significant differences from controls could only be demonstrated if the data for 1 to 7 days were pooled ($P < 0.01$).

*(d) Combined treatment:* Six to 12 mice were used per dose-point. The same four intervals of 1, 7, 21 and 28 days examined above, were used for the measurement of $ED_{50}$ values for hyperthermia or PDT, following a first tolerance treatment of either 194 J $cm^{-2}$ of light or 40 min heating at 43°C. In the combined modality experiments, drug was given 1 day before light, and intervals quoted are for 'light–hyperthermia' or 'hyperthermia–light'. Two sets of experiments were carried out: the first a comparison of 1 day and 7 days, when for both modalities the $^{133}Xe$ T½ values differed markedly; the second set a comparison of 7, 21 and 28 days, for which the T½ values were more closely similar. Comparing 1 and 7 days, whether tolerance PDT or hyperthermia was the first treatment, the $ED_{50}$ for the second agent was significantly lowered relative to the $ED_{50}$ for that agent given 'alone' (i.e. with a sham first treatment; Table 1). The 1 days interval led to a slightly greater reduction than 7 days, but the difference was insignificant. The second set of experiments confirmed that 7 days after a first treatment, the tissue remained markedly sensitised to the second agent, that by 28 days this sensitisation had largely disappeared, but that as long as 21 days after the combination 'PDT–hyperthermia', residual sensitisation might persist.

**Figure 3** Areas occupied by various tissue compartments in cross-sections of tails in animals either untreated (□), treated by hyperthermia (■) or by PDT (▨). Error bars are 1SE for inter-animal variation,

### Discussion

The environmental conditions under which PDT and hyperthermia exert their maximal effect, are very different. Photodynamic therapy with drugs such as HPD, requires the presence of molecular oxygen and thus usually an intact blood supply. Conversely, interruption of the vasculature enhances the damaging effects of hyperthermia, because the cooling property of an intact blood flow is lost. These differences were readily seen in the present model. In common however, the *expression* of tumour or tissue damage both by PDT and hyperthermia, is mediated in large part by vascular injury (Star *et al.*, 1986; Reinhold & Endrich, 1986). The model demonstrated clearly the interference both by PDT and hyperthermia, with vascular function as measured by rates of clearance of locally-injected radioisotope. A decrease in $^{133}Xe$ clearance rate was observed 10 min after the completion of PDT or hyperthermia (50–80 min after the start of treatments; Figures 2, 5). Previous isotope studies of responses of skin to hyperthermia, using as an assay the percentage extraction by tissue of systemically injected $^{86}Rb$, have commonly shown an early *increase* in 'flow' (e.g. Stewart & Begg, 1983; Song *et al.*, 1987). However the temperature–time combinations used in our study were deliberately high, tissue tolerance or greater, and Song *et al.* (1987) noted in their series, that after the more severe temperature regimens (e.g. 44.5°C for 60 min, 'in air'), blood flow 1–5 h after heating mouse leg skin might be reduced to a level only half that of controls. Such observations raise an apparent paradox, because one possible consequence of this reduced perfusion after HT might be to lower tissue oxygenation, in which case one would expect subsequent PDT at short intervals (e.g. at 1 day, Figure 2) to be ineffective, and yet it is seen that this interval is *most* effective in reducing $ED_{50}$ for PDT (Table I). Firstly, it should be noted that even $ED_{50}$ doses of HT do not cause complete abrogation of blood flow ($^{133}Xe$ T½ for controls = 2 min, for HT a maximum of 7 min, 60 min on the application of a clamp). The most recent estimate made *in vitro* of the $O_2$ concentration at which PDT becomes less than maximally effective gives the very low value of $< 0.1\%$ (after a 24 h exposure to the HPD-related compound Photofrin II; Chapman *et al.*, 1991). Also it should be noted that using the present model, the most important parameter determining the probability of necrosis is not a very high acute value for T½ but a prolonged

**Figure 4** Absolute numbers of blood vessels in the hypodermis of cross-sections of tails, in animals either untreated (▨), or treated by hyperthermia (HT) or PDT (■). Error bars are 1SE for inter-animal variation.

**Table I** $ED_{50}$ values for PDT or hyperthermia, either given alone, or at varying intervals after a first treatment by a 'tolerance' dose of the other agent (HPD plus 194 J $cm^{-2}$ for PDT, and 40 min at 43°C for clamped hyperthermia).

| *Experiment No.* | *First agent* | *Interval (days)* | $ED_{50}$ *for Second agent*[a] |
|---|---|---|---|
| 1 | | | HEAT |
| | None, or light alone | – | 50 ± 1 min |
| | PDT | 1 | 32 ± 2 min[b] |
| | PDT | 7 | 34 ± 3 min[b] |
| | | | PDT |
| | Sham-heated | – | 351 ± 50 J $cm^{-2}$ |
| | HEAT | 1 | 184 ± 7 J $cm^{-2b}$ |
| | HEAT | 7 | 199 ± 20 J $cm^{-2b}$ |
| 2 | | | HEAT |
| | None, or light alone | – | 54 ± 2 min |
| | PDT | 7 | 44 ± 4 min[b] |
| | PDT | 21 | 44 ± 3 min[b] |
| | PDT | 28 | 55 ± 2 min |
| | | | PDT |
| | Sham-heated | – | 333 ± 45 J $cm^{-2}$ |
| | HEAT | 7 | 128 ± 39 J $cm^{-2b}$ |
| | HEAT | 21 | 301 ± 12 J $cm^{-2}$ |
| | HEAT | 28 | 306 ± 54 J $cm^{-2}$ |

[a]Error as 1 SE for inter-animal variation.
[b]Significantly different from values for single agents ($P<0.05$).

**Figure 5** Exponential half-time of clearance from the tail of $^{133}$Xe, as a function of time after a tolerance dose (▲) or an $ED_{50}$ dose (■) if PDT. All symbols, errors, as for Figure 2.

*duration* of sub-optimal perfusion (compare $ED_5$ and $ED_{50}$ in Figure 2; and see Benstead & Moore, 1988a).

The marked acute effects of PDT and hyperthermia that have been observed here, manifested by a transient swelling of tissue compartments (Figure 3), are consistent with the oedematous, inflammatory response that has been described after PDT (e.g. for mouse ear, (Lim *et al.*, 1986) or hyperthermia (e.g. for mouse foot; Wondergem & Haveman, 1984). In contrast to hyperthermia, the onset of oedema after PDT required a few days for full development (Figure 3), which confirms the results of previous measurements of gross tail volume in our system, using the sensitiser di-haemato-porphyrin 'ether' (Moore *et al.*, 1986). Onset may be site-related: in the mouse ear and using HPD, Lim *et al.* (1986) found a peak swelling at 24 h, resolving thereafter.

As regards vascular function, in a previous study using this tail model and the photosensitiser $TPPS_4$, clearance inhibition was directly associated with the probability of skin necrosis when light dose was delivered as two fractions separated by increasing intervals of days (Benstead & Moore, 1988b). In that study therefore, improvement in $^{133}$Xe clearance was associated with 'recovery' processes that led to tissue sparing. Our expectation was that, judging by the qualititative similarities between PDT and HT with respect to $^{133}$Xe clearance (Figures 2, 5), we might see sparing by increasing the interval between these modalities, particularly between 1 and 7 days. This expectation was not met. The marked enhancement of skin damage was the same at both intervals, whichever was the first agent given (Table I). This enhancement was unexpected in that it has been claimed that PDT, like HT, produces 'heat shock proteins', that *in vitro* at least, cause a loss of interaction between the two modalities (Mang & Dougherty, 1985), and 'thermal tolerance' leading to murine skin sparing (most marked between 1 and 3 days) has been demonstrated following split HT schedules of tolerance plus test doses (Law *et al.*, 1979). Large changes in probability of necrosis did occur over the subsequent period (7 to 28 days) during which time however, $^{133}$Xe clearance varied little ($T\frac{1}{2}$ being shorter than in controls; Figs 2, 5). This non-association of clearance time of $^{133}$Xe and probability of necrosis, using the lipophilic sensitiser HPD is in seeming contrast with our earlier results using the hydrophilic sensitiser $TPPS_4$ (Benstead & Moore, 1988*b*). The possibility must be considered that the effects on a given vasculature of light in combination with different photosensitisers, may vary. Thus the peak degree of oedema induced by tolerance $TPPS_4$ plus light was only 60% that induced with HPD (Table 1 in Benstead and Moore (1989), Figure 3, this paper). Also, 5 days after PDT with $TPPS_4$ the absolute number of capillaries per tail circumference was 60% greater than in controls, whereas with HPD there was no significant increase in the number of small vessels relative to age-matched controls (Figure 5 in Benstead & Moore (1989), Figure 4, this paper). An observation requiring further investigation is that although absolute and relative numbers of vessels returned to control levels by 21/28 days (Figure 4), $^{133}$Xe clearance time remained significantly lower than controls (Figures 2, 5), suggesting functional differences in vessels 'recovering' after these therapies.

A closer association was found between quantitative vascular histology and the probability of necrosis, in that during the first 7 days there was a marked reduction in the proportion of the smallest vessels, i.e. those participating in nutrient exchange with the parenchyma, and a concomitant increase in larger-bore vessels (Figure 4). Only with the restoration toward the normal relationships of the different-sized vessels by 21 or 28 days, was the enhancement of injury abrogated. It may be significant that, using the mouse foot model and a 'tolerance' dose of hyperthermia (44°C for 60 min), Haveman *et al.* (1988) observed the onset of granulation tissue at 7 days, within which was a highly proliferative endothelium, and a progressively higher proliferation index in the basal cells of the dependent epidermis between 7 and 21 days.

In summary, this paper has demonstrated that an increased incidence of injury occurred in a normal tissue, skin, when hyperthemia and photodynamic therapy were given in close temporal association, that such enhancement persisted for at least one week, and that sparing of the tissue (i.e. such that the probability of injury was no more than that to be expected for a given dose of either modality alone) could be achieved by further separating the treatments by 2–3 weeks. It appears important when combinations of HT and PDT using HPD are advocated for the treatment of tumours, that the consequences for surrounding normal tissues also be assessed.

Ms. P. Nuttall and colleagues in the Regional Medical Physics Department, Christie Hospital, kindly supplied equipment, isotope and advice for xenon clearance studies. The authors' work is supported by the Cancer Research Campaign (UK).

### References

BENSTEAD, K. & MOORE, J.V. (1988*a*). Vascular function and the probability of skin necrosis after photodynamic therapy: An experimental study. *Br. J. Cancer*, **57**, 451.

BENSTEAD, K. & MOORE, J.V. (1988*b*). The effect of fractionation of light treatment on necrosis and vascular function of normal skin following photodynamic therapy. *Br. J. Cancer*, **58**, 301.

BENSTEAD, K. & MOORE, J.V. (1989). Quantitative histological changes in murine tail skin following photodynamic therapy. *Br. J. Cancer*, **59**, 503.

CHAPMAN, J.D., STOBBE, C.C., ARNFIELD, M.R., SANTUS, R., LEE. J. & MCPHEE, M.S. (1991). Oxygen dependency of tumour cell killing in vitro by light-activated Photofrin II. *Radiat. Res.*, **126**, 73.

DE RUTIER, J. & VAN PUTTEN, L.M. (1975). Measurement of blood flow in the mouse tail after irradiation. *Radiat. Res.*, **61**, 427.

GLATSTEIN, E. (1973). Alterations in rubidium-86 extraction in normal mouse tissues after irradiation. *Radiat. Res.*, **53**, 88.

HAVEMAN, J., JANSEN, W., WONDERGEM, J. & BEGG, A.C. (1988). Cell proliferation in the murine epidermis and subcutaneous vascular endothelium after hyperthermia. *Int. J. Radiat. Biol.*, **54**, 105.

HENDRY, J.H. (1978*a*). Mouse rail radionecrosis. In Streffer, C. (ed) *Cancer Therapy by Hyperthermia and Radiation*, Urban and Schwarzenberg: Baltimore, p. 216.

HENDRY, J.H. (1978*b*). Radionecrosis of normal tissue: Studies on mouse tails. *Int. J. Radiat. Biol.*, **33**, 47.

LAW, M.P., COULTAS, P.G. & FIELD, S.B. (1979). Induced thermal resistance in the mouse ear. *Br. J. Radiol.*, **52**, 308.

LEVENDAG, P.C., MARIJNISSEN, H.P.A., DE RU, V.J., VERSTEEG, J.A.C., VAN RHOON, G.C. & STAR, W.M. (1988). Interaction of interstitial photodynamic therapy and interstitial hyperthermia in a rat rhabdomyosarcoma–a pilot study. *Int. J. Radiat. Oncol. Biol. Phys.*, **14**, 139.

LIM, H.W., HAGAN, M. & GIGLI, I. (1986). Phototoxocity induced by haematoprophyrin derivative in C5-deficient, mast-cell deficient and leukopaenic mice. *Photochem. Photobiol.*, **44**, 175.

MANG, T.S. & DOUGHERTY, T.J. (1985). Time and sequence dependent influence of *in vitro* photodynamic therapy (PDT) survival by hyperthermia. *Photochem. Photobiol.*, **42**, 533.

MOORE, J.V., KEENE, J.P. & LAND, E.J. (1986). Dose-response relationships for photodynamic injury to murine skin. *Br. J. Radiol.*, **59**, 257.

REINHOLD, H.S. & ENDRICH, B. (1986). Tumour microcirculation as a target for hyperthermia. *Int. J. Hyperthermia*, **3**, 535.

SONG, C.W., PATTEN, M.S., CHELSTROM, L.M., RHEE, J.G. & LEVITT, S.H. (1987). Effect of multiple heatings on the blood flow in RIF-1 tumours, skin and muscle of CH mice. *Int. J. Hyperthermia*, **3**, 535.

STAR, W.M., MARIJNISSEN, H.P.A., VAN DEN BERG-BLOK, A.E., VERSTEEG, J.A.C., FRANKEN, K.A.P. & REINHOLD, H.S. (1986). Destruction of rat mammary tumour and normal tissue microcirculation by haematoprophyrin derivative photoradiation observed *in vivo* in sandwich observation chambers. *Cancer Res.*, **46**, 2532.

STEWART, F.A. & BEGG, A. (1983). Blood flow changes in transplanted mouse tumours and skin after mild hyperthermia. *Br. J. Radiol.*, **56**, 477.

WALDOW, S.M., HENDERSON, B.W. & DOUGHERTY, T.J. (1987). Hyperthermic potentiation of photodynamic therapy employing Photofrin I and II; Comparison of results using three animal tumour models. *Lasers Surg. Med.*, **7**, 12.

WONDERGEM, J. & HAVEMAN, J. (1984). A study of the effects of prior heat treatment on the skin reaction of mouse feet after heat alone or combined with X-rays: influence of misonidazole. *Radiother. Oncol.*, **2**, 159.